# Prevention is better than cure

**DOI:** 10.4103/0972-0707.53332

**Published:** 2009

**Authors:** S Ramachandran

**Affiliations:** Ragas Dental College and Hospital, Chennai, India E-mail: ramachandran_principal@yahoo.co.in

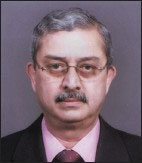



Conservative dentistry is a dynamic discipline. Changes and advancements have taken place with respect to the techniques, materials, concepts, and the technology in the past few decades. Knowledge of these changes and the techniques used in diagnosing and treating the world's most prevalent disease - the dental caries - has been the goal for our specialty. Dental caries is now recognized as an infectious disease; hence it is important for us to understand the underlying cause for dental caries, and to clinically intervene surgically or nonsurgically to prevent the disease. The restoration of a carious lesion no longer is considered a cure. Rather, the clinician must identify patients who have active carious lesions and patients at high risk for caries, and institute appropriate preventive and treatment measures.

Caries preventive methods include fluoride treatment, immunization, salivary functioning, antimicrobical agents, diet, oral hygiene, xylitol gums, and sealent application. Caries control methods include operative procedures of removing the irreversibly damaged tooth, pulpal therapy, and the restoration of the damaged tooth surface to appropriate anatomic contours and function. Prevention is still the least costly alternative treatment option.

It goes without saying that there is no research without education. The academic teams for research in various institutions should aim in developing quality diagnostic tools and better preventive and control measures for dental caries. I think we being the specialists in the field should focus on the diagnostic and preventive services and embrace new and accepted dental treatments, materials, and devices for the benefit of the patient and work toward improving the standard of dental treatment in our country.

I consider it an honor for having been invited to share my views in this column and I wish the JCD team all the best.

